# The gut microbiota as a potential determinant of response to GLP-1 receptor agonists: a narrative review

**DOI:** 10.3389/fendo.2026.1858420

**Published:** 2026-07-06

**Authors:** Luigi Regenburgh De La Motte, Francesca Carreras, Lorenzo Drago

**Affiliations:** 1 Unità Operativa Complessa UOC Laboratory of Clinical Medicine with Specialized Areas, Istituto di Ricovero e Cura a Carattere Scientifico IRCCS MultiMedica, Milan, Italy; 2 Clinical Microbiology and Microbiome Laboratory, Department of Biomedical Sciences for Health, University of Milan, Milan, Italy

**Keywords:** GLP-1 receptor agonists, gut microbiome modulation, incretin–microbiota axis, metabolism, therapeutic response variability

## Abstract

Interindividual variability in clinical response to glucagon-like peptide-1 receptor agonists (GLP-1 RAs) represents a significant challenge in the management of obesity and type 2 diabetes (T2D). In recent years, growing interest has focused on the potential role of the gut microbiota as a biological modifier of incretin-based therapy. This narrative review synthesizes current preclinical and human evidence on the bidirectional interactions between GLP-1 RAs and the intestinal microbial ecosystem, with particular attention to microbial composition, metabolite production (including short-chain fatty acids and bile acids), intestinal barrier integrity, and inflammatory signaling. Experimental models consistently demonstrate that GLP-1 RAs can remodel gut microbial communities and influence metabolite profiles. In human studies, GLP-1 RA therapy has been associated with changes in microbial diversity and enrichment of specific taxa; however, most available data remain observational and associative. Small exploratory cohorts suggest that baseline microbiota composition may correlate with differential metabolic response, giving rise to the responder/non-responder framework. Nevertheless, definitions of response are heterogeneous, study populations are limited in size, and mechanistic causality has not been established. Importantly, microbiota changes observed during GLP-1 RA therapy may be influenced by confounding factors such as weight loss magnitude, dietary modifications, and concomitant treatments, particularly metformin. Functional pathway inferences frequently rely on 16S rRNA-based predictions rather than direct metabolomic measurements, warranting cautious interpretation. Overall, current evidence supports the hypothesis that host–microbiome interactions may contribute to therapeutic heterogeneity, but robust longitudinal and interventional human studies are required before microbiome-informed stratification or adjunctive microbiota-targeted interventions can be considered for clinical implementation. Elucidating these interactions may ultimately refine precision approaches to incretin-based therapy in metabolic disease.

## Introduction

**Figure 1:**
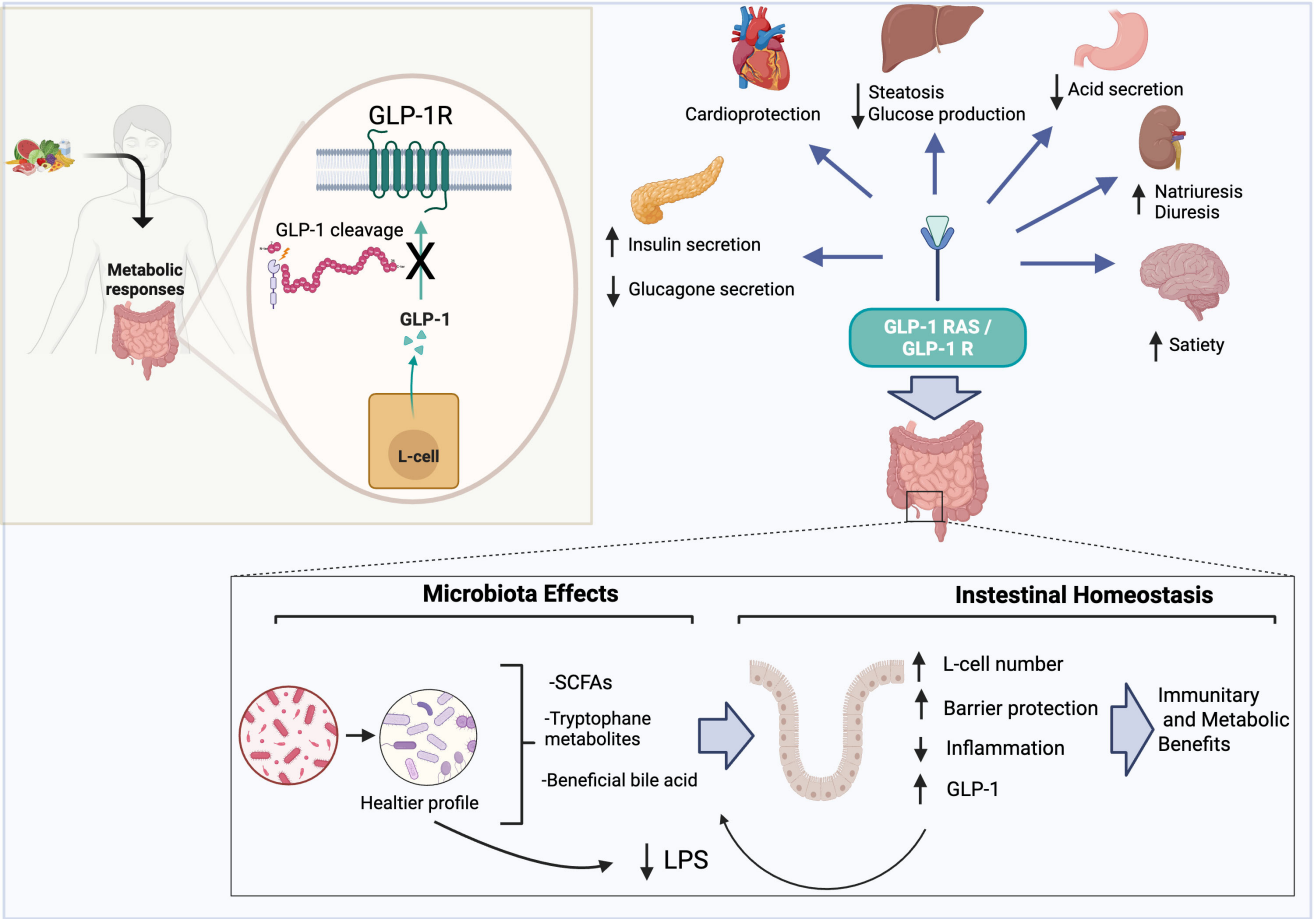
Overview of GLP-1/GLP-1R actions and their interaction with the gut microbiota. After stimulation by luminal nutrients, intestinal L cells release GLP-1, which, similarly to GLP-1 receptor agonists (GLP-1 RAs), acts through widely distributed GLP-1 receptors to regulate multiple physiological systems (pancreas, liver, heart, kidney, stomach, and brain). The metabolic and endocrine effects of GLP-1/GLP-1R signaling on insulin secretion, glucagon suppression, appetite regulation, and cardiometabolic outcomes are supported by robust experimental and clinical evidence. Interactions involving the gut microbiota-including modulation of microbial composition, production of short-chain fatty acids and bile acid metabolites, reinforcement of intestinal barrier integrity, and potential influence on therapeutic heterogeneity are supported primarily by preclinical studies and associative human data. These pathways represent biologically plausible mechanisms that may contribute to metabolic regulation but remain incompletely established in humans. Created with https://www.BioRender.com.

Glucagon-like peptide-1 receptor agonists (GLP-1 RAs) are a key therapeutic class in the management of T2D and obesity, where they not only improve glycemic control and promote weight loss, but also exert significant effects on the gastrointestinal system. GLP-1 is an incretin hormone produced by the intestinal L-cells in response to nutrient stimulation (1, 2). It enhances postprandial insulin secretion in a glucose-dependent manner and binds to the GLP-1 receptor (GLP-1R), a class B1 G-protein-coupled receptor widely expressed across multiple organs, including the endocrine pancreas, gastrointestinal tract, heart, kidneys, lungs, central nervous system, adipose tissue, immune cells, and even chondrocytes, the primary cells of articular cartilage (1). As illustrated in **Figure 1**, this broad anatomical distribution of GLP-1R enables GLP-1 and its receptor agonists to modulate multiple interconnected metabolic and neuroendocrine pathways. Native GLP-1 has a short half-life due to rapid degradation by dipeptidyl peptidase-4 (DPP-4) (3). In contrast, GLP-1 RAs are engineered for DPP-4 resistance and prolonged exposure; moreover, GLP-1 receptors are expressed in brain regions involved in appetite regulation, supporting central contributions to their metabolic effects (4). This has made GLP-1R an attractive pharmacological target for incretin-based therapies. GLP-1 RAs mimic the physiological actions of GLP-1 by activating its receptor, leading to reduced glucagon secretion, enhanced insulin release, delayed gastric emptying, and improved glucose homeostasis (5). Their ability to lower HbA1c while inducing significant weight reduction has positioned them as highly effective treatments for obesity and metabolic dysfunction (6).

In recent years, growing evidence has highlighted a bidirectional interplay between GLP-1 RAs and the gut microbiome. Metabolic disorders such as obesity and diabetes are frequently associated with gut dysbiosis, characterized by reduced microbial diversity, loss of beneficial taxa, and increased inflammatory signatures, which contributes to insulin resistance and metabolic impairment (7–10). GLP-1 RAs have been shown to modulate the gut bacterial composition, partially restoring eubiotic profiles and influencing microbial metabolites linked to metabolic health (2, 11). Conversely, the resident microbiota and its metabolites can influence the host’s response to GLP-1 RA therapy, a complex gut–hormone axis with potential implications for personalized treatment (12, 13).

Approved GLP-1 RAs include exendin-4–based agents (exenatide, lixisenatide) and human GLP-1 analogues such as liraglutide, semaglutide, and dulaglutide. In addition, dual incretin agonists (e.g., GIP/GLP-1 co-agonists such as tirzepatide) have expanded this therapeutic space, while other long-acting or multi-agonist candidates remain investigational (14).

This review provides a structured overview of how GLP-1 RAs impact gut microbiota composition and function, drawing on recent human clinical studies and mechanistic animal models. We further explore whether microbiome-targeted strategies, including probiotics, prebiotics, and postbiotics, may modulate or enhance these effects. Particular emphasis is placed on key microbial-host pathways such as short-chain fatty acid (SCFAs) production, bile acid metabolism, intestinal barrier integrity, immune signaling, and the gut–brain axis.

While recent high-quality reviews have extensively covered the general crosstalk between GLP-1 signaling and the gut microbiota, or focused broadly on pharmacomicrobiomics in metabolic diseases, a comprehensive critical evaluation centered on therapeutic heterogeneity remains scarce. This narrative review uniquely addresses the emerging ‘responder vs. non-responder’ framework. Specifically, we dissect how baseline microbial signatures correlate with differential efficacy, while critically assessing clinical confounding factors, such as metformin co-treatment and dietary shifts, and the methodological limitations that currently hinder the translation of microbiome profiling into precision endocrinology.

## Literature search strategy

This article is a narrative review. A structured literature search was conducted using PubMed and Scopus databases up to January 2026. The search strategy included combinations of the following keywords: “GLP-1 receptor agonists”, “GLP-1 RA”, “gut microbiota”, “microbiome”, “short-chain fatty acids”, “bile acids”, “intestinal barrier”, “inflammation”, and “interindividual variability”.

Studies were selected based on their relevance to the interactions between GLP-1 receptor agonists and the gut microbiota, with particular attention to mechanisms potentially influencing treatment response and metabolic outcomes. Priority was given to human studies, randomized controlled trials, prospective observational studies, and high-quality mechanistic preclinical investigations. Recent publications and seminal studies in the field were preferentially included. Reference lists of relevant articles were also screened to identify additional studies of interest.

Articles not directly related to the review topic, studies lacking sufficient methodological detail, and reports providing redundant information were not prioritized for inclusion.

No formal PRISMA methodology, risk-of-bias assessment, or evidence grading system was applied, as the aim of this work was to provide a critical narrative synthesis of the current evidence rather than a systematic quantitative assessment. Conclusions were drawn by considering the consistency of findings across studies and giving greater weight to evidence derived from human clinical investigations whenever available.

Furthermore, this review primarily focuses on the bacterial component of the gut microbiome, which represents the most extensively investigated domain in relation to GLP-1 receptor agonist therapy. Although emerging evidence suggests potential roles for the gut virome and mycobiome in metabolic regulation, these components remain insufficiently characterized within the context of GLP-1 RA treatment and are therefore beyond the scope of the present review.

## Effects of GLP-1 RAs on gut microbiota: preclinical and clinical evidence

The gut microbiota plays a central role in metabolic regulation, particularly in lipid and energy homeostasis, and its alteration is increasingly recognized as a risk factor for obesity, T2D and cardiovascular disease (15, 16). Dietary changes profoundly affect microbial composition; for example, high-fat diet (HFD) models show shifts in the relative abundance of major phyla such as *Bacillota* (formerly *Firmicutes*), *Bacteroidota* (formerly *Bacteroidetes)*, and *Proteobacteria*, with obesity-associated patterns often reflecting decreased microbial diversity and loss of beneficial taxa (17–19). In both rats and humans, T2D has been associated with reduced levels of butyrate-producing bacteria and overall dysbiosis, which may contribute to insulin resistance and systemic inflammation (20). In this context, several microbiota-derived metabolites, including SCFAs, trimethylamine (TMA), bile acids, coprostanol, phenylacetylglutamine, and lipopolysaccharide (LPS), have gained attention due to their established involvement in cardiovascular diseases (21).

Given this evidence, targeting the gut microbiota has emerged as a potential strategy for preventing or ameliorating metabolic diseases, including T2D and dyslipidemia (22). Importantly, several studies suggest that specific microbial communities, particularly *Bacillota* and *Bacteroidota*, may influence insulin sensitivity partly through modulation of GLP-1 secretion. Indeed, SCFAs derived from bacterial fermentation of dietary fibers reaching the distal intestine are thought to trigger the secretion of the incretin hormone GLP-1, as demonstrated by mixed colon cultures *in vitro (*23). Because GLP-1 activity is closely intertwined with intestinal microbial ecology, interventions that enhance GLP-1 signaling can, in turn, remodel the gut microbiota. Indeed, GLP-1 RAs, a drug class originally developed for glycemic control and more recently approved for weight management, have demonstrated the ability to influence gut microbial composition. These agents exert well-known cardiometabolic benefits (24), and their potential to modulate host–microbe interactions is an area of rapidly expanding scientific interest. Meanwhile, the emerging field of pharmacomicrobiomics seeks to determine how the gut microbiota affects drug absorption, distribution, metabolism, excretion (ADME), and toxicity; this includes GLP-1 RAs, whose efficacy may be partially shaped by the host microbial environment.

Together, these observations provide the rationale for examining how GLP-1 RAs alter the gut microbiota in controlled experimental models. Below, we summarize key findings from preclinical animal studies, followed by human clinical evidence, to delineate how GLP-1–microbiota interactions may contribute to the therapeutic actions of this drug class.

### GLP-1 receptor agonists and the gut microbiome: evidence from experimental models

Preclinical models consistently demonstrate that GLP-1 RAs can reshape the gut microbiota composition toward a “healthier” profile (25). Liraglutide is among the most extensively studied GLP-1 RAs in preclinical models of obesity. Most mechanistic evidence derives from preclinical models, while human data remain largely associative. In diet-induced obese or diabetic rodents, GLP-1 analogs like liraglutide have been shown to increase the relative abundance of beneficial bacteria while reducing potentially harmful taxa. For example, a 2022 study in HFD-fed mice showed that 12-week liraglutide treatment markedly reduced the relative abundance of *Bacillota*, while increasing *Bacteroidota*, resulting in a marked decrease in the *Bacillota/Bacteroidota* ratio. This shift was accompanied by enrichment of numerous SCFA-producing and mucin-degrading genera such as *Akkermansia*, *Lactobacillus*, *Parabacteroides*, and *Oscillospira (*26). The same treatment suppressed several pro-inflammatory or opportunistic microbial taxa, including *AF12*, *Shigella*, members of the phylum *Proteobacteria*, and *Xenorhabdus*. These shifts correlated with improvements in metabolic parameters, notably, reduced body weight and improved dyslipidemia (lowered LDL-C and total cholesterol), suggesting that modulation of the gut microbiota is one mechanism by which GLP-1 RAs confer metabolic benefits and lipid-lowering effect (26). Indeed, in obese mice it was seen that antibiotic-induced depletion of *Bacillota* and *Bacteroidota* alters the pool of intestinal metabolites, in particular by increasing taurocholic acid, which in turn stimulates the release of GLP-1 from L cells, improving glucose tolerance (19). Treatment with liraglutide altered the gut microbiota community in diabetic male rats, leading to an increase in *Bacteroides*, members of the family *Lachnospiraceae*, and the beneficial genus *Bifidobacterium*, which was linked to lowered intestinal inflammation and improved glycemic control (27). In obese and diabetic rats, liraglutide enhances carbohydrate and lipid metabolism while profoundly remodeling the gut microbiota, reducing obesity-associated taxa (such as *Romboutsia, Ruminiclostridium*, and *Erysipelotrichaceae*) and enriching lean mass-related genera (e.g., *Prevotella*), with an increase in the *Bacteroidota* -*Bacillota* ratio and a clear correlation between microbial changes and metabolic parameters. The same result was observed in obese non-diabetic rats, indicating that the weight-controlling effect of liraglutide was independent of glycemic status (28). Although liraglutide and saxagliptin, a DPP-4 inhibitor, both act on the incretin axis, in animal models liraglutide exerted a markedly superior microbiota modulating effect, profoundly restructuring the architecture of the intestinal community. Specifically, liraglutide reduced phylotypes associated with obesity (e.g., *Roseburia, Erysipelotrichaceae Incertae Sedis, Marvinbryantia, Parabacteroides*) and enriched genera related to thinness (e.g. *Blautia, Coprococcus*), while these changes were absent with saxagliptin, consistent with its neutral effect on body weight (29). In addition, liraglutide and semaglutide improved reproductive and metabolic disorders while modulating the overall structure of the gut microbiota in mice with polyendocrine metabolic ovarian syndrome (PMOS) (30); formerly polycystic ovary syndrome (PCOS), liraglutide increased butyrate-producing *Lachnospiraceae* and partially reversed dysbiosis, while semaglutide uniquely increased *Helicobacter*, the only taxon negatively correlated with body weight (31). In HFD-fed mice, an increase in gut microbiota alpha diversity was observed based on the ACE, Chao, Simpson, Shannon, and Sobs indices. All indices, except for the Simpson index, which remained unchanged, were elevated after liraglutide treatment at both low and high doses compared with untreated HFD-fed mice. This pattern indicates that liraglutide enhances microbial diversity, with the effect being particularly pronounced at the lower dose (32). In diabetic kidney disease rats treated with liraglutide, an increase in the Simpson diversity index was observed together with marked improvements in renal tubular structure, demonstrating that the renoprotective effect of liraglutide was linked to enhancements in gut microbiota composition; notably, 5-OP (5-Oxoproline) levels were positively correlated with *Clostridium* abundance and negatively correlated with renal injury markers, further supporting a microbiota-mediated mechanism (33). Notably, GLP-1 RAs also tend to promote growth of genera known for supporting gut barrier integrity (such as *Akkermansia*), further hinting at a positive remodeling of the gut ecosystem (34, 35).

Interpretation of preclinical microbiome findings should consider the inherent limitations of rodent models. Important differences from humans include the disproportionately large cecum, coprophagic behavior, standardized laboratory diets, and specific-pathogen-free housing conditions, all of which can substantially influence microbial composition and metabolic interactions. Consequently, direct translation of microbiome-related findings from rodents to humans should be approached with caution.

### GLP-1 receptor agonists and the gut microbiome: evidence from human studies

In humans, the impact of GLP-1 RAs on fecal microbiota composition has been more variable, with results influenced by treatment duration and inter-individual differences.

Short-term clinical trials have sometimes reported minimal or no significant shifts in gut microbiota despite marked metabolic improvements. Most studies investigating the effects of GLP-1 RAs on the human gut microbiota have been conducted in patients with T2D.

For instance, in a clinical randomized trial of 52 patients with T2D, 16S rRNA profiling showed that 6 weeks of liraglutide (or colesevelam) did not produce discernible changes in gut microbiota composition, even though patients experienced altered bile acid profiles (36).

However, another study in diabetic patients showed functional genetic predictions indicating that liraglutide might increase microbial genes associated with improved carbohydrate and lipid metabolism, suggesting a shift toward a healthier functional capacity of the gut microbiota (37). Since dysbiosis is characterized not only by altered microbial composition but also by loss of key metabolic functions, these changes may reflect a partial restoration of microbial metabolic activity despite modest taxonomic shifts. Similarly, a preliminary longitudinal study in newly diagnosed T2D patients found no acute microbiota change one week after starting dulaglutide; only after 48 weeks of treatment did significant alterations emerge, including an overall reduction in bacterial abundance and shifts in specific taxa. This delayed effect suggests that long-term metabolic remodeling (and possibly dietary changes following weight loss) may be required to observe microbiome changes in humans. Indeed, after 48 weeks on dulaglutide, patients showed a markedly different community structure compared to baseline, with certain bacterial populations decreasing in relative abundance while others increased, indicating that sustained GLP-1 RA therapy can reshape the gut microbiota over time (38).

Other human studies have reported microbiota changes concordant with those seen in animal models. An observational study of obese T2D patients with concurrent MASLD (metabolic dysfunction-associated steatotic liver disease) showed that 12 weeks of liraglutide significantly increased gut bacterial diversity and shifted the community toward a profile more like healthy individuals (39). In this study, liraglutide-treated patients saw an uptick in the relative abundance of *Bacteroidota* and *Proteobacteria* (class *Bacilli* also increased), whereas an active comparator (metformin) enriched different taxa (*Fusobacteria* and *Actinobacteria*) and had a larger effect on network connectivity of the microbiota. Interestingly, liraglutide’s effects on the gut microbial network were “weaker” than metformin’s, resulting in a post-treatment microbiota composition that more closely resembled that of healthy controls (suggesting a normalization of dysbiosis rather than a drastic reconfiguration) (39).

Another study of 52 subjects with T2D treated with GLP-1 RAs found that responders exhibited higher abundances of beneficial species such as *Bacteroides dorei, Roseburia inulinivorans, Lachnoclostridium* sp.*, and Butyricicoccus* sp., whereas non-responders showed enrichment of taxa such as *Prevotella copri*, a microbe associated with insulin resistance, and several members of *Ruminococcaceae* and *Alistipes*. In this pilot study, patients were classified as responders and non-responders according to the magnitude of HbA1c reduction after GLP-1 RA therapy. After 12 weeks of treatment, gut microbiome β-diversity diverged significantly between responders and non-responders, and multivariate analyses confirmed that baseline levels of *B. dorei*, *Lachnoclostridium*, and *Mitsuokella* independently predicted glycemic response to GLP-1 RA therapy. These findings highlight a bidirectional relationship: GLP-1 RAs can modulate the gut microbiota, while the pre-existing microbial configuration may influence therapeutic efficacy and reflect the degree of dysbiosis in T2D (40).

More recent real-world analyses on gut microbiome fecal samples of patients with T2D.

further suggest that semaglutide may impact the gut microbial community during treatment, and the basal microbiome may predict changes in HbA1c in semaglutide users (despite methodological limitations and the need for replication) (41).

Consistent with these findings, a recent clinical pilot study further demonstrated that liraglutide not only promoted weight reduction and improved glycemic control in patients with obesity and T2D, but also induced significant shifts in gut microbial composition (α and β diversity) and metabolic pathways, reinforcing the concept that microbiota modulation may contribute to the therapeutic effects of GLP-1 RAs (42).

To synthesize the currently available human evidence, **Table 1** summarizes clinical studies evaluating gut microbiota composition in patients receiving GLP-1 receptor agonists. Across these investigations, most studies report therapy-associated microbial compositional changes, whereas only a limited number have specifically explored baseline microbiome features associated with treatment response. Overall, the available evidence remains limited by small cohorts, methodological heterogeneity, and predominantly associative study designs.

**Table 1:** Human clinical studies evaluating gut microbiota changes and microbial features associated with GLP-1 receptor agonist therapy.

Study	Population	GLP-1 RA	Microbiome method	Key microbial findings	Association with clinical response	Major limitations
(37)	T2D	Liraglutide	16S rRNA	Altered microbiota structure and reduced alpha diversity after treatment	Changes associated with metabolic parameters	Small sample size
(40)	T2D	Liraglutide/Dulaglutide	16S rRNA	Distinct microbial signatures between responders and non-responders	Baseline taxa predicted glycemic response	Pilot study
(38)	T2D	Dulaglutide	16S rRNA	Long-term therapy alters microbial composition	Correlations with HbA1c and BMI	No responder analysis
(39)	T2D + MASLD	Liraglutide vs Metformin	16S rRNA	Increased microbial diversity and altered taxa	Associated with metabolic improvement	Small cohort
(41)	T2D	Semaglutide	16S rRNA	Baseline microbiome predicts treatment response	Predictive association with HbA1c change	Very small sample
(42)	T2D	GLP-1RA + DMR	Shotgun sequencing	Microbial diversity correlated with HbA1c and liver fat	Associations with metabolic improvement	Combined intervention
(36)	BAD patients	Liraglutide	16S rRNA	No significant microbiota change	No association with response	Non-diabetic population

Summary of human clinical studies investigating gut microbiota composition in patients receiving GLP-1 receptor agonists. Most available studies report therapy-associated microbial compositional changes, whereas only a limited number specifically evaluate baseline microbiome features associated with treatment response. The evidence is limited by small cohorts, heterogeneous study designs, and variable microbiome methodologies.

### Comparative effects among GLP-1 RAs on microbiota

Different GLP-1 RA drugs (liraglutide, exenatide, dulaglutide, semaglutide) appear to have broadly similar trends in microbiota modulation, though nuances exist. A recent systematic review (2025) concluded that liraglutide generally promotes the growth of metabolically beneficial taxa, consistent with improved host metabolic functions (43). Although liraglutide-induced changes in gut microbiota composition are heterogeneous across studies, consistent enrichment of metabolically beneficial taxa, particularly *Akkermansia muciniphila, Lactobacillus*, and *Bifidobacterium*, along with a reduction of obesity-associated genera emerges as a reproducible pattern, highlighting that species-level and functional alterations are more informative than phylum-level shifts (27, 44).

In human gut microbiota analyses, one study reported increases in *Faecalibacterium prausnitzii* and *Peptostreptococcus anaerobius (Bacillota)*, as well as *Bacteroides vulgatus (Bacteroidota) and Akkermansia muciniphila (Verrucomicrobiota*) *(*45). After treatment with dulaglutide, mice showed significant β-diversity differences compared with saline controls, characterized by increased *Bacteroidota* and reduced *Bacillota*, a microbial profile associated with lower insulin resistance and obesity; similar shifts were also observed after empagliflozin and combined treatment (46). In animal studies, exenatide (and its analogue exendin-4) increased microbes tied to better metabolism (47). Human data on exenatide remain limited and somewhat inconsistent; however, one study reported that exenatide treatment was associated with selective gut microbiota changes, predominantly at the species level, including increases in potentially beneficial taxa such as *Bifidobacterium*, *Coprococcus*, and *Akkermansia muciniphila*, as well as several butyrate-producing species (e.g., *Roseburia hominis* and *R. intestinalis*), together with reductions in specific *Bacillota* genera (48). Importantly, exenatide-induced microbiota changes were functionally linked to inflammation and glycemic control, with enrichment of *Akkermansia* and taxa correlating negatively with inflammatory markers, and reductions in pathogenic bacteria correlating with improved metabolic profiles (43, 49). Moreover, in preclinical studies, semaglutide treatment was associated with an increased abundance of several gut microbial taxa, including *Bacteroides, Muribaculaceae, Alloprevotella, Alistipes, Blautia, Dubosiella, Enterococcus, Allobaculum, Clostridia_UCG-014, Coriobacteriaceae_UCG-002*, and notably *Akkermansia*, suggesting a shift toward a microbiota profile enriched in taxa linked to metabolic regulation (31, 49–51).

Notably, oral semaglutide introduces a unique pharmacomicrobiomics paradigm compared to subcutaneous formulations. Formulated with the absorption enhancer sodium N-(8-[2-hydroxybenzoyl]amino)caprylate (SNAC), oral semaglutide undergoes direct mucosal exposure in the stomach (52). This localized delivery and high gastric concentration may exert direct, proximity-dependent effects on the upper gastrointestinal microbiota and local epithelial barrier integrity before systemic absorption, a feature completely absent in injectable regimens.

Supporting this concept, a recent preclinical study demonstrated that chronic exposure to the absorption enhancer SNAC significantly altered gut microbial community structure, reduced the abundance of saccharolytic bacteria and fecal butyrate levels, and was associated with changes in inflammatory markers, suggesting that components of the oral formulation itself may influence host–microbiome interactions independently of systemic GLP-1 receptor activation (53).

Crucially, the expanding landscape of incretin mimetics requires a clear distinction between standard mono-agonists and next-generation or multi-agonist formulations. Tirzepatide, a dual GIP/GLP-1 receptor co-agonist, may induce a specific microbial footprint that differs from pure GLP-1 agonism, as GIP signaling independently participates in lipid handling and mucosal immune regulation, potentially modifying the nutrient-delivery profile to the gut (54). Indeed, recent study demonstrated that tirzepatide partially restored gut microbiota homeostasis in mice with diabetic nephropathy, improving microbial balance and attenuating the progression of diabetic kidney disease (55).

Overall, differences between studies likely reflect heterogeneity in patient populations, diet, baseline microbiota, and treatment duration. Nonetheless, the common theme is that GLP-1 RAs tend to push the microbiome in a direction associated with metabolic health (greater abundance of SCFA-producing and mucus-associated bacteria, lower abundance of pro-inflammatory organisms), although substantial changes may require prolonged therapy (56).

## Microbiota-derived metabolites and gut hormone signaling

### SCFAs and GLP-1 crosstalk

The gut microbiota and its derived metabolites play a central role in modulating host metabolism, immune function, insulin sensitivity, and overall health (57).

Among these metabolites, SCFAs (primarily acetate, propionate, and butyrate) act as critical signaling molecules linking microbial fermentation with host energy regulation. Emerging evidence suggests that the baseline metabolic capacity of the gut microbiota to ferment these dietary substrates may represent a crucial upstream biological factor contributing to the interindividual heterogeneity observed in clinical outcomes. Specifically, it is hypothesized that the presence of a functional and diverse reservoir of SCFA-producing taxa at baseline supports a more receptive endocrine environment. In individuals exhibiting robust therapeutic outcomes, these microbial metabolites potentially initiate a complementary signaling cascade via free fatty acid receptors on enteroendocrine L-cells, thereby reinforcing host incretin pathways. Conversely, a pre-existing restriction in this fiber-fermenting machinery could blunt this supportive postbiotic feedback loop, offering a plausible microbial rationale for the sub-optimal metabolic responses observed in non-responders (40, 58).

GLP-1 RA treatment has been reported to influence microbial composition in experimental models, potentially favoring fiber-fermenting bacteria (58). However, most of the evidence supporting this interaction derives from preclinical studies, and direct measurements of SCFA production in humans receiving GLP-1 RAs remain limited. Beyond serving as energetic substrates, SCFAs exert important endocrine functions by binding to G-protein–coupled receptors, namely FFAR2 (GPR43) and FFAR3 (GPR41), expressed on enteroendocrine L cells and other cell types across rodents and humans (59, 60). Activation of GPR43 by SCFAs triggers intracellular signaling cascades, including increases in intracellular calcium concentration (59, 61) and induces L-cells to secrete endogenous GLP-1 and peptide YY (PYY) (62–64).

GPR43 (FFAR2) shows higher sensitivity to acetate and propionate than to butyrate, whereas GPR41 is primarily activated by propionate and butyrate (65, 66). Notably, propionate has been shown to directly stimulate GLP-1 release in controlled experimental settings, creating a positive feedback loop whereby GLP-1 RA-induced microbial changes lead to more SCFA production, which in turn further amplifies endogenous GLP-1 and PYY secretion (67). Whether this feedback mechanism operates to a clinically meaningful extent in humans treated with GLP-1 RAs remains to be clarified. Consistently, GLP-1 RA therapy is associated with higher circulating levels of PYY in humans, an appetite-suppressing hormone often co-secreted with GLP-1, and this may be partly mediated by SCFA signaling (58, 63). SCFAs (especially butyrate and propionate) also interact with the autonomic nervous system and hypothalamus to promote satiety and improve insulin sensitivity in peripheral tissues (68).

In metabolic disorders such as T2D and obesity, a dysbiotic, low-SCFA microbiome, often characterized by a higher Bacillota-to-Bacteroidota ratio and a depletion of Akkermansia muciniphila, is associated with impaired baseline incretin secretion, contributing to insulin resistance (69–72).

By favoring the enrichment of SCFA-related taxa, GLP-1 RAs may enhance the availability of these beneficial postbiotics, reinforcing host metabolic homeostasis through gut–brain and gut–endocrine pathways (68, 73).

This reciprocal reinforcement may partly explain the superior metabolic outcomes observed when GLP-1 RA therapy is combined with a healthy, fiber-rich diet that supports SCFA-producing microbes and endogenous incretin signaling (7, 23, 74). However, in humans, these relationships remain predominantly inferential and require confirmation through longitudinal and mechanistically designed studies incorporating direct metabolomic measurements.

Beyond direct cellular signaling, the primary pharmacological actions of GLP-1 RAs, namely, the significant delay in gastric emptying and the alteration of overall gastrointestinal transit time, exert profound physiological pressure on the luminal environment (75). By slowing transit, these agents fundamentally alter the rate and spatial distribution of nutrient and substrate delivery to the distal gut. This extended retention time modifies luminal pH gradients and local oxygen availability, shifting the competitive dynamics between saccharolytic and proteolytic fermentation (76, 77). Consequently, these physiological changes create distinct physiological niches that shape microbiota colonization patterns and metabolic outputs, operating independently of any direct, receptor-mediated drug–microbiota interactions.

### Bile acid–microbiota–GLP-1 interactions

Variability in therapeutic responsiveness may be functionally linked to baseline differences in microbial bile acid metabolism. The relative abundance of bacterial genera possessing high bile salt hydrolase (BSH) activity, such as Akkermansia and Bacteroides, varies significantly among individuals, potentially modifying the host’s secondary bile acid pool prior to treatment initiation (78, 79). Indeed, gut bacteria play a central role in this metabolic framework by expressing BSH and downstream enzymes that deconjugate primary bile acids (synthesized from cholesterol in the liver) and transform them into secondary bile acids, profoundly modifying their physicochemical properties, intestinal reabsorption, and receptor signaling (80). An efficient baseline enzymatic transformation of these molecules may yield a distinct ligand profile capable of co-activating host farnesoid X receptor (FXR) and G protein-coupled bile acid receptor TGR5 pathways, thereby synergizing with the exogenous drug to improve metabolic flexibility (80). In contrast, a dysbiotic microbial configuration characterized by reduced BSH functional potential may contribute to a less efficient microbial transformation of bile acids, potentially influencing the composition of the host bile acid pool and its downstream signaling through FXR and TGR5 pathways. This altered baseline metabolic context could modulate the host response to GLP-1 receptor agonist therapy, which has itself been associated with microbial remodeling and shifts in gut-derived signaling metabolites (81).

Obesity and HFD are associated with perturbations in bile acid homeostasis, including increased hepatic bile acid synthesis and altered circulating and intestinal bile acid profiles (82). These alterations contribute to metabolic dysregulation by modulating lipid absorption, glucose metabolism, and inflammatory signaling. Increasing evidence suggests that GLP-1 RAs, beyond their direct endocrine actions, can indirectly influence bile acid metabolism through modulation of the gut microbiota (56, 82). GLP-1 RA treatment has been associated with enrichment of bacterial genera such as *Akkermansia* and *Bacteroides*, which include species with bile salt hydrolase activity and actively participate in bile acid deconjugation (83, 84).

Microbiota-driven changes in deconjugation and downstream transformation can modify the physicochemical properties of bile acids and their reabsorption in the distal ileum, thereby potentially affecting enterohepatic recycling and the relative availability of specific bile acid species within the intestinal lumen (82, 85, 86). Rather than implying clinically relevant fat malabsorption, these shifts are more plausibly interpreted as alterations in bile acid signaling and metabolic handling of nutrients that may complement the appetite-suppressive and insulin-sensitizing actions of GLP-1 RAs (86, 87). Beyond their digestive role, bile acids function as signaling molecules by activating host receptors such as FXR and the G protein-coupled bile acid receptor TGR5, integrating microbial activity with host glucose and lipid metabolism (88, 89). Through these combined mechanisms, GLP-1 RAs may contribute to remodeling of bile acid pools both directly and indirectly via microbiota interactions, thereby reinforcing gut-liver endocrine crosstalk and metabolic homeostasis (56, 87).

### Gut barrier integrity and inflammation

The functional culmination of these microbial shifts, particularly the enrichment of SCFA-producing taxa and BSH-active bacteria, is the preservation of gut barrier integrity and the reduction of systemic low-grade inflammation. In this context, the heterogeneity of responses to GLP-1 RAs therapy supports a responder–non-responder framework that may be partly driven by baseline differences in microbial functional capacity and host–microbiome inflammatory status.

In obesity and T2D, the depletion of beneficial taxa allows the expansion of potentially harmful, endotoxin-producing bacteria beyond physiological equilibrium (90–92). This dysbiotic state, strongly promoted by a HFD, compromises the intestinal mucus layer and weakens tight junction integrity, leading to increased intestinal permeability (‘leaky gut’) (90, 93, 94).

The resulting translocation of bacterial lipopolysaccharides (LPS) and other pro-inflammatory molecules into the portal and systemic circulation triggers chronic low-grade inflammation, which directly exacerbates peripheral insulin resistance and can disrupt vagal afferent signaling within the gut–brain axis, thereby impairing physiological appetite control (95, 96).

There is strong evidence that intestinal inflammation, such as that observed in inflammatory bowel disease (IBD), involves dysregulation of epithelial immune responses to the gut microbiota (97–99).

Consistent with this, the depletion of butyrate-producing bacteria and the concomitant reduction in intestinal butyrate levels reported in IBD patients compared with healthy individuals have highlighted the immunomodulatory role of this microbial metabolite (91, 100–102).

GLP-1 RAs help break this pathogenic cycle by fostering a mucosal environment that supports structural health. At the epithelial level, the drug-induced expansion of butyrate-producing bacteria becomes structurally pivotal: butyrate serves as the primary fuel for colonocytes, stimulates tight junction assembly (upregulating proteins such as occluding and zonula occludens-1 [ZO-1]), and induces mucin production from goblet cells, thereby thickening the protective mucus layer. Concurrently, a higher abundance of Akkermansia muciniphila supports adipose tissue insulin sensitivity via extracellular vesicle signaling dependent on Toll-like receptor 2 (TLR2) (93, 100).

Animal models treated with GLP-1 RAs demonstrate a significant reduction in plasma LPS levels and attenuated intestinal tissue inflammation, reflecting a combined effect of a restored microbiota and direct intestinal GLP-1R signaling (103, 104).

In parallel, GLP-1 RAs exert direct immunomodulatory effects on immune cells. For example, exendin and exenatide, two GLP-1 RAs, have been shown to suppress macrophage secretion of multiple pro-inflammatory cytokines, including IFN-γ, IL-17, IL-2, TNF-β, IL-6, and IL-1β (103, 105). Indeed, one study in patients with T2D receiving GLP-1 RA therapy reported that individuals with a higher abundance of *Faecalibacterium prausnitzii* in the gut microbiota exhibited better glycemic control and lower levels of systemic inflammation, supporting a link between GLP-1–based treatment, beneficial microbial signatures, and metabolic–inflammatory outcomes (106). Improved gut barrier integrity also prevents the leakage of microbial products that can trigger adipose and hepatic inflammation. In obese mice, liraglutide was shown to reverse HFD-induced increases in gut permeability and reduce inflammatory cytokine expression in intestinal tissues (35). These effects reflect a combination of restoration of a more favorable gut microbiota and direct GLP-1R signaling within the intestine. The net result is a reduction in systemic endotoxin burden and inflammatory tone. As inflammation is attenuated, insulin signaling in peripheral tissues improves, and weight loss is facilitated, given that chronic inflammation can promote leptin resistance and dysregulated appetite control.

While much of this is due to weight loss, the gut-mediated component is increasingly recognized. By enhancing gut barrier function, GLP-1 RAs cut off a major source of inflammation, the leaky gut, thus helping to resolve metabolic inflammation (107). This also has implications for the gut–brain axis because a more intact barrier and lower inflammation allow neural and hormonal signals of satiety to be transmitted more effectively (96). Collectively, these observations support the view that inter-individual variability in GLP-1 RA efficacy may, at least in part, reflect differences in baseline gut barrier integrity and microbiome functional capacity, which may distinguish responders from non-responders.

## Microbiota-based interventions to enhance GLP-1 RA efficacy (probiotics, prebiotics, postbiotics)

Given the bidirectional interplay between GLP-1 RAs and the gut microbiota, increasing attention has been directed toward microbiota-targeted strategies to address key clinical challenges, such as inter-individual response variability and substantial weight regain after treatment discontinuation. Probiotics (live beneficial microorganisms), prebiotics (selectively fermentable substrates), and postbiotics (microbial-derived metabolites or functional components) represent complementary approaches aimed at improving gut microbial composition and function, thereby potentially reinforcing incretin signaling and metabolic outcomes (108).

Evidence suggests that modulating the microbiota influences glucose homeostasis through endogenous GLP-1 secretion and intestinal barrier function. A recent systematic review of oral probiotic interventions in metabolic disorders reported modest but consistent improvements in glycemic parameters, including fasting glucose and HbA1c, alongside signals of enhanced incretin activity, supporting a mechanistic link between probiotic supplementation and gut hormone regulation (109).

From a mechanistic standpoint, specific beneficial commensal taxa most notably species within *Lactobacillus*, *Bifidobacterium*, and selected butyrate-producing anaerobes, have been shown to increase SCFA production, strengthen epithelial barrier integrity, and stimulate enteroendocrine L cells (110). These effects converge on pathways already targeted by GLP-1 RAs, suggesting a potential synergistic interaction rather than redundancy (43).

Prebiotic supplementation may further potentiate these effects by selectively enriching beneficial microbial populations. Fermentable fibers such as inulin, fructooligosaccharides, and resistant starch promote the expansion of SCFA-producing bacteria, leading to increased acetate, propionate, and butyrate availability. These metabolites are known to stimulate GLP-1 and PYY secretion and improve gut barrier function, processes that may enhance the metabolic efficacy of GLP-1 RA therapy when combined with dietary fiber enrichment (2, 56).

The clinical relevance of such complementary approaches is highlighted by evidence showing that co-administration of probiotics, such as *Limosilactobacillus fermentum* GB102, with GLP-1RAs (e.g., dulaglutide) significantly enhances weight loss and attenuates weight regain after treatment cessation in obese mice (111). Furthermore, GB102 preserves muscle strength, a critical factor often compromised during pharmacologically induced weight loss. Although these findings remain limited to preclinical models, they support the concept that microbiota-targeted interventions may complement GLP-1 RA therapy beyond the period of active pharmacological treatment. This interaction underscores a broader metabolic complementarity: while GLP-1 RAs primarily drive weight loss through appetite suppression, delayed gastric emptying, and insulinotropic effects, microbiota-targeted interventions may act on additional pathways involved in energy expenditure, substrate utilization, and muscle preservation, thereby potentially reducing inter-individual variability in therapeutic outcomes.

While probiotics and prebiotics aim to reshape microbial communities and their metabolic activity, postbiotics offer an alternative strategy by directly delivering bioactive microbial products, particularly in individuals characterized by marked dysbiosis or reduced microbial diversity. The direct administration of microbiota-derived metabolites, such as short-chain fatty acids (SCFAs) and indole derivatives, has been shown in experimental models to improve insulin sensitivity, reduce inflammation, and promote gut hormone secretion, potentially bypassing the need for sustained microbial engraftment (112).

Notably, some of the beneficial effects observed with *L. fermentum* GB102 may be mediated by its metabolic products. GB102 is characterized by a distinctive metabolic signature associated with high succinate production (111). Succinate is a microbiota-derived metabolite that has been linked to the modulation of host energy metabolism, including the activation of thermogenic pathways in brown adipose tissue (113). More broadly, a growing body of preclinical evidence suggests that certain postbiotic preparations may attenuate weight gain, reduce adiposity, and stimulate thermogenesis, including brown adipose tissue signaling, particularly in HFD-induced rodent models (114). These effects appear to be mediated through improvements in insulin sensitivity, modulation of lipid metabolism, and activation of gene programs involved in enteroendocrine function and thermogenesis (115–117).

However, the translation of these findings to human obesity remains limited by important physiological differences between rodents and humans, including the lower abundance and activity of brown adipose tissue in adults and an overall reduced thermogenic capacity. Therefore, in the current clinical context, postbiotics may be more realistically positioned not as primary weight-loss interventions but rather as complementary nutritional strategies to be implemented following the discontinuation of GLP-1 receptor agonist therapy (118). In this setting, they may contribute to maintaining metabolic stability, limiting weight regain, and preserving lean body mass during the post-treatment phase.

## Microbiota signatures of response and non-response to GLP-1 RA therapy

Interindividual variability in therapeutic response to GLP-1 RAs has sparked growing interest in the role of the gut microbiota in determining drug efficacy. Beyond established pharmacological mechanisms such as improved glucose-dependent insulin secretion, delayed gastric emptying, and appetite modulation, growing evidence indicates that the intestinal microbial ecosystem may contribute to interindividual differences in treatment outcomes. Specific microbial taxa, functional pathways, and host–microbe interactions have been associated with differential metabolic responses to GLP-1 RA therapy (84). However, current evidence remains largely observational and exploratory. Understanding these microbiota signatures may generate hypotheses for future precision approaches, but does not yet support clinical stratification.

One of the most informative human studies in this field is one pilot trial, which demonstrated that patients achieving robust glycemic improvements following GLP-1 RA therapy displayed a distinct baseline microbial profile characterized by greater α-diversity and enrichment of SCFA–producing bacteria, including *Roseburia inulinivorans*, *Lachnoclostridium* spp., *Butyricicoccus*, and *Faecalibacterium prausnitzii* (40). These taxa contribute to butyrate and propionate production, metabolites that have been implicated in modulating endogenous GLP-1 secretion, intestinal barrier integrity, and inflammatory tone. Nevertheless, the observed associations do not establish a causal relationship between specific taxa and glycemic response. A summary of the gut microbiota features associated with differential response to GLP-1 RA therapy in human studies is provided in **Table 2**. The same study highlighted higher baseline abundance of *Bacteroides dorei* in responders, suggesting that certain *Bacteroides* species may be associated with improved metabolic flexibility or incretin responsiveness. Additional taxa enriched among non-responders, including *Alistipes* spp. and selected members of the *Ruminococcaceae* family, have similarly been associated with pro-inflammatory or dysmetabolic states, although their role in modulating GLP-1 RA efficacy remains speculative.

**Table 2:** Microbial taxa and community features reported in responders and non-responders to GLP-1 RA therapy.

Microbiota feature	Non-responder pattern (vs responders)	Metabolic implication	Reference
β- diversity (PCoA)	Partial separation from responders, with significant directional clustering (p = 0.004)	Divergent community structures associated with differential metabolic response	(39)
Relative abundance of key taxa	↓Bacteroides dorei (p< 0.001); enrichment of Prevotella copri, Ruminococcaceae sp., Bacteroidales sp., and other dysbiosis-linked taxa	Reduced representation of taxa commonly linked to SCFA production and microbial signatures associated with poorer HbA1c reduction following GLP-1 RA therapy	

Summary of gut microbiota characteristics associated with differential response to GLP-1 RA treatment. The table highlights differences in community structure (β-diversity) and relative abundance of specific bacterial taxa observed between responders and non-responders, together with their potential metabolic implications. PCoA, principal coordinates analysis; SCFA, short-chain fatty acid.

Conversely, non-responders exhibited lower microbial diversity and a predominance of taxa associated with metabolic impairment, most notably *Prevotella copri*. The metabolic relevance of *P. copri* is supported by evidence showing that this species is a major driver of branched-chain amino acid (BCAA) biosynthesis and is strongly linked to insulin resistance in humans (119). Elevated BCAA levels have been linked to impaired insulin signaling and reduced metabolic flexibility. However, whether *P. copri* abundance directly modulates GLP-1 RA responsiveness remains unproven.

These host–microbe interactions have been extensively reviewed, highlighting a bidirectional regulatory axis in which GLP-1 signaling and microbial metabolites coordinate energy balance and immune–metabolic processes. However, definitions of clinical response differ across studies, complicating cross-study comparisons. Standardization of microbiome analytics and response criteria will be essential to improve reproducibility and enable meaningful meta-analytic integration. In this review, the terms response, responder, and non-responder are used as defined in the original studies (most commonly HbA1c reduction and/or weight loss, when reported, over the study-specific follow-up), given the lack of standardized response criteria across the literature.

In addition, microbial functional profiles characterized by increased representation of LPS biosynthesis and branched-chain amino acid metabolism-pathways previously linked to insulin resistance and low-grade inflammation, have been hypothesized to contribute to reduced metabolic responsiveness. However, these inferences are often derived from predictive functional analyses based on 16S rRNA sequencing rather than direct metabolomic measurements, and direct clinical validation in GLP-1 RA-treated cohorts remains limited (56).

Preclinical studies provide mechanistic evidence for interactions between the gut microbiota and incretin biology, which could suggest potential relevance to GLP-1 RA response. In particular, experimental studies have demonstrated that manipulation of the gut microbiota can influence endogenous GLP-1 secretion and metabolic outcomes, indicating a mechanistic role for host–microbe interactions under controlled preclinical models (19). For example, liraglutide and semaglutide have been shown to remodel the gut microbiota toward SCFA-producing and mucus-associated taxa in rodent models (43). Similarly semaglutide has been shown to improve metabolic parameters in obese mice while increasing microbial diversity and enriching *Akkermansia muciniphila*, a mucin-degrading bacterium associated with improved gut barrier function and reduced inflammation (120). Although these models do not distinguish responders from non-responders, they reinforce the concept that GLP-1 RAs interact dynamically with the gut microbial ecosystem and that the magnitude of microbial remodeling may shape clinical outcomes. While these findings support biological plausibility, their translational relevance to human responder phenotypes remains uncertain.

Notably, similar concepts have emerged from studies on metformin, where interindividual variability in therapeutic response and gastrointestinal tolerability has been linked to differences in gut microbiota composition. In this context, microbiota-targeted interventions such as fecal microbiota transplantation have been proposed as complementary strategies to restore microbial diversity and improve metabolic outcomes, highlighting the broader relevance of host–microbiome interactions in shaping antidiabetic drug efficacy (9).

Baseline microbial profiling may eventually contribute to understanding variability in GLP-1 RA response, but its routine use for therapeutic stratification is not supported by current evidence.

Variability in GLP-1 RA response is multifactorial and cannot be attributed to any single determinant (121). Rather, host-related characteristics (e.g., baseline metabolic status, inflammatory burden, genetic background and biological sex), environmental influences such as diet and caloric intake, and pharmacological factors including drug dose, duration, and concomitant therapies collectively shape treatment outcomes (41, 122–126). Considering the broader genetic background, pharmacogenomic variability may further contribute to treatment heterogeneity, as genetic variants affecting GLP-1 receptor signaling, appetite regulation, insulin secretion, and host–microbiota interactions could influence individual responsiveness (126).

**Figure 2:**
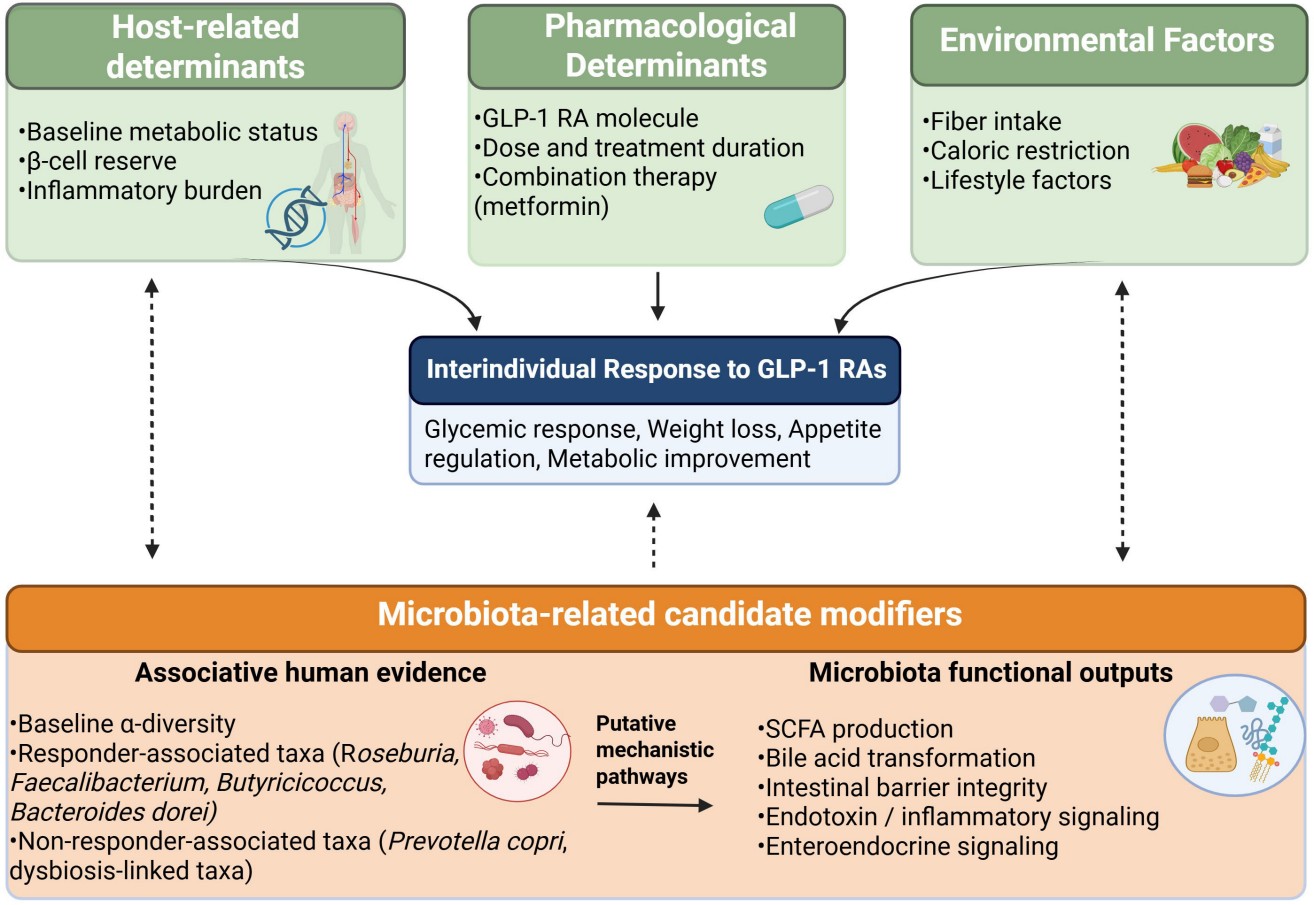
Conceptual model of factors contributing to interindividual variability in GLP-1 receptor agonist response. Established determinants include host-related characteristics, environmental influences, and pharmacological factors, which directly impact therapeutic outcome. Bidirectional dashed arrows indicate biologically plausible interactions between the microbiota, host, and environmental factors, while the dashed connection with treatment response reflects emerging, but not yet clinically validated, evidence.

Biological sex is increasingly recognized as a relevant determinant, as sex-specific differences in hormone profiles, immune function, gut microbiota composition and GLP-1 RA pharmacodynamics have been associated with differences in weight-loss outcomes and gastrointestinal tolerability (127). Importantly, these determinants are interdependent and may amplify or attenuate one another within a complex host-microbe-drug network (128). Within this context, the gut microbiota can be considered a potential component of this integrated system, given its involvement in enteroendocrine and incretin signaling; however, current human evidence remains largely associative and does not allow causal inference. To provide a structured overview of factors consistently reported in the literature, these determinants are summarized in **Table 3**, while their interplay and the positioning of microbiota-related features within this multifactorial context are conceptually illustrated in **Figure 2**.

**Table 3:** Overview of factors associated with interindividual variability in GLP-1 RA response.

Variability domain	Representative determinants	Reference
Host-related factors	Baseline BMI, insulin resistance severity, β-cell function, high baseline HbA1c, inflammatory status, genetics/pharmacogenomic, biological sex	(122, 124, 126, 127, 129)
Microbial factors	Baseline α-diversity, enrichment of SCFA-producing taxa, Prevotella copri abundance, inferred pro-inflammatory functional signatures	(40, 41, 128)
Environmental factors	Dietary composition, caloric intake	(130, 131)
Pharmacological factors	GLP-1 RA dose, duration, combination with metformin	(121, 125, 132)

Representative host-related, microbial, environmental, and pharmacological determinants that have been associated with variability in GLP-1 RA treatment outcomes across preclinical and clinical studies. BMI, body mass index; HbA1c, glycated hemoglobin; SCFA, short-chain fatty acid.

## Discussion

Accumulating evidence suggests that the relationship between GLP-1 RAs and the gut microbiome may extend beyond a simple secondary consequence of weight loss or glycemic improvement, although the relative contribution of direct pharmacological effects versus indirect metabolic changes remains incompletely defined. In addition to established endocrine mechanisms, such as glucose-dependent insulin secretion, delayed gastric emptying, and appetite suppression, GLP-1 RAs engage in complex bidirectional interactions with the intestinal ecosystem (58). Within the gut–brain framework, enteroendocrine cells act as chemosensory sentinels that translate luminal cues into endocrine and paracrine signals detected by vagal afferents, providing a concrete route through which microbiota-derived signals could modulate neuroendocrine circuits relevant to therapeutic heterogeneity (16, 68).

Across both preclinical models and human observational studies, GLP-1 RA therapy has frequently been associated with shifts toward a more eubiotic microbial configuration. Reported changes include enrichment of short-chain fatty acid (SCFA)–producing taxa, increased representation of bile acid–modifying bacteria, and relative reductions in Gram-negative, endotoxin-producing organisms (133).

Microbiota-derived metabolites represent a plausible mechanistic interface: SCFAs and secondary bile acids can strengthen epithelial barrier function, modulate host metabolism via FXR- and TGR5-dependent signaling, and attenuate low-grade inflammation, potentially reinforcing pharmacologically targeted pathways.

Small exploratory human studies have reported associations between baseline microbial composition and differential metabolic outcomes. Individuals classified as responders often exhibit higher microbial diversity and greater relative abundance of SCFA-producing and anti-inflammatory taxa, including *Roseburia*, *Faecalibacterium*, and *Butyricicoccus*. In contrast, non-responders more frequently display dysbiotic profiles enriched in taxa linked to insulin resistance and inflammatory metabolism, such as *P. copri (*39). Notably, several microbial features observed in non-responders overlap with taxa previously associated with greater T2D severity and pro-inflammatory metabolic states (70, 134). This suggests that reduced responsiveness to GLP-1 RA therapy may, at least in part, reflect a more advanced or entrenched dysbiotic state rather than a direct failure of incretin signalingThis framework places microbiota-related factors within a hierarchical network of host, environmental, and pharmacological determinants, emphasizing their role as modifiers rather than primary drivers of therapeutic variability.

Rather than providing a purely descriptive account of microbial shifts, we propose a conceptual framework in which interindividual variability arises from a multi-level, interactive network of determinants.

At the primary level, host-related factors, including baseline metabolic status, insulin resistance, β-cell function, inflammatory burden, and genetic background, represent the dominant drivers of therapeutic response. These factors define an individual baseline metabolic response potential upon which pharmacological interventions act. At a second level, environmental and treatment-related variables, such as dietary composition, caloric intake, weight loss dynamics, and concomitant pharmacological therapies, modulate this baseline response. These determinants are dynamic and may influence both metabolic outcomes and gut microbial composition during therapy. Within this hierarchy, the gut microbiota act as an integrated modulatory layer rather than a independent predictor; its functional outputs (SCFA production, bile acid transformation, and barrier integrity) amplify or attenuate host responses through dynamic, bidirectional feedback loops.

Collectively, these observations underscore the potential relevance of intestinal microbial homeostasis in metabolic disease management (135). Recurrent associations between dysbiotic microbial patterns and poor metabolic response further support the hypothesis that gut microbiota may contribute to interindividual differences in response to incretin-based therapies, although definitive mechanistic evidence in humans remains limited (136), and causality between specific microbial taxa, functional pathways, and GLP-1 RA responsiveness cannot be inferred.

The most common adverse events associated with GLP-1 receptor agonists are gastrointestinal symptoms, particularly nausea and vomiting, whereas pancreatitis represents a rare but clinically relevant concern. While GLP-1 receptor agonists have been shown to influence gut microbiota composition, evidence linking microbiota characteristics to susceptibility to these adverse events remains limited. Furthermore, no convincing evidence currently supports a direct association between gut microbiota composition and the risk of GLP-1 receptor agonist-associated pancreatitis.

Future longitudinal and interventional studies integrating microbiome profiling, metabolomics, and controlled designs will be required to determine whether specific microbial configurations can act as clinically actionable modifiers of GLP-1 receptor agonist response.

### Methodological limitations and confounding factors

Despite these promising associations, a rigorous critique of current literature reveals substantial methodological and clinical limitations that hinder translation. First, functional pathway alterations in human trials frequently rely on computational inferences derived from 16S rRNA sequencing rather than direct shotgun metagenomics sequencing or transcriptomic measurements. These predictive models should be interpreted cautiously and not equated with confirmed metabolic outputs.

Second, important differences among GLP-1 receptor agonists themselves remain insufficiently explored. Most microbiome studies have focused on liraglutide, semaglutide, and dulaglutide, whereas evidence regarding short-acting agents such as exenatide remains comparatively limited. Given the substantial differences in pharmacokinetics, duration of receptor exposure, effects on gastric emptying, and weight-loss efficacy among individual compounds, drug-specific microbiota effects are highly plausible but currently masked by underpowered, heterogeneous study designs.

Third, the extent to which major clinical confounding factors have been adequately controlled remains highly uncertain. Weight loss itself independently increases diversity, while mandatory dietary modifications accompanying therapy represent major determinants of microbial structure (137, 138). Concomitant pharmacological treatments, particularly metformin, further complicate interpretation due to their documented microbiota-modulating effects. Metformin has been associated with increased abundance of *Akkermansia muciniphila* and other mucin-degrading or SCFA-producing bacteria, as well as with alterations in intestinal GLP-1 secretion and circulating lipopolysaccharide (LPS) levels. These changes have been linked to improvements in insulin sensitivity and metabolic parameters, potentially through modulation of the gut–pancreas hormonal axis (139). As many patients receiving GLP-1 RAs are treated with combination regimens, attribution of observed microbial shifts exclusively to GLP-1 RAs should be approached cautiously. Rigorous adjustment for weight loss magnitude, dietary intake, and co-medications is frequently lacking in current human studies. To provide a structured overview of these potential confounders, **Table 4** summarizes whether the main human studies included in this review reported or adjusted for weight loss, dietary factors, and metformin use. Overall, formal adjustment for these variables was uncommon, highlighting an important source of heterogeneity across studies.

**Table 4:** Assessment of major confounding factors across human studies investigating the effects of GLP-1 receptor agonists on the gut microbiota.

Study	Weight loss reported/adjusted	Dietary assessment/adjustment	Metformin adjustment	Comments
(37)	Reported through BMI/HbA1c changes, not formally adjusted	Not reported	Controlled by design: metformin was replaced by liraglutide	Metformin-to-liraglutide substitution design
(40)	BMI change reported, not formally adjusted	Assessed by validated FFQ, not formally adjusted	Antidiabetic medications recorded, no formal adjustment	Responder/non-responder pilot study
(38)	BMI measured longitudinally, not formally adjusted	Standardized inpatient diet; continuation monitored	Excluded at baseline	Longitudinal dulaglutide study
(39)	BMI/weight reported, not formally adjusted	Unified dietary and exercise guidance; no formal adjustment	Comparator arm included metformin	Liraglutide versus metformin
(41)	BMI/waist reported, not formally adjusted	Not reported	Controlled by design: all participants on stable metformin	Predictive microbiome analysis
(42)	Body weight reported, not formally adjusted	Post-procedural diet and nutritional counselling provided	Oral glucose-lowering drugs continued at stable dose	Combined DMR and GLP-1 RA intervention
(36)	Not primary endpoint	Not reported	Not applicable	Non-diabetic BAD population

Summary of the extent to which key clinical confounders were reported or controlled in human studies evaluating GLP-1 receptor agonist–associated microbiota changes. Variables assessed include weight loss, dietary factors, and metformin exposure, all of which may independently influence gut microbiota composition and function. BMI, body mass index; FFQ, food frequency questionnaire; DMR, duodenal mucosal resurfacing.

An additional knowledge gap concerns pediatric and adolescent populations, which remain substantially underrepresented in studies evaluating interactions between GLP-1 receptor agonists and the gut microbiome. Given the increasing use of incretin-based therapies in younger individuals with obesity and metabolic disorders, future studies should investigate whether microbiota-related determinants of treatment response differ across developmental stages and age groups.

### A structured roadmap for clinical translation

From a translational perspective, microbiome-based stratification cannot be recommended as a routine clinical tool due to limited human evidence, methodological heterogeneity, absence of standardized analytical pipelines, and lack of validated microbial cut-offs. Although a substantial subset of patients exhibits suboptimal metabolic responses to therapy, precise prevalence estimates of non-responders remain uncertain, and no randomized controlled trials have demonstrated that microbiome-guided selection improves GLP-1 RA outcomes.

Interest has therefore emerged in adjunctive microbiome-targeted strategies, including probiotics, prebiotics, and postbiotics, as potential means to enhance GLP-1 RA efficacy or mitigate gastrointestinal adverse effects.

Nonetheless, evidence from randomized controlled trials investigating fecal microbiota transplantation (FMT) supports the translational plausibility of microbiome-targeted interventions. A recent meta-analysis of RCTs reported improvements in several metabolic parameters, including markers of glycemic control, insulin sensitivity, and lipid metabolism, demonstrating that targeted modulation of the gut ecosystem can shift clinically relevant endpoints in humans (140). Crucially, beyond its potential therapeutic application to prevent post-discontinuation weight regain, fecal microbiota transplantation (FMT) must be positioned as an indispensable mechanistic research tool. In translational science, FMT provides a unique experimental platform to transcend purely associative human data; by transplanting fecal communities from clinically verified responder or non-responder human phenotypes into germ-free rodent models, investigators can systematically isolate and verify the causal contribution of specific microbial configurations to incretin drug efficacy.

Moving from mechanistic observations to clinical implementation will require a structured translational roadmap:

1. Methodological Standardization: Standardizing microbiome sampling, sequencing platforms (transitioning from 16S rRNA to high-resolution shotgun metagenomics), and bioinformatic pipelines to improve reproducibility across international cohorts.
2. Functional Validation: Integrating taxonomic profiling with direct untargeted metabolomics to validate biologically relevant pathways (such as true SCFA and bile acid quantification) beyond predictive signatures.
3. Prospective Covariate Control: Designing adequately powered prospective longitudinal studies capable of testing whether baseline microbial signatures predict treatment outcomes while strictly controlling for diet, metformin use, and weight loss dynamics.
4. Interventional Agnostic Trials: Conducting randomized clinical trials to evaluate whether targeted adjunctive microbiota modulations (probiotics, prebiotics, or postbiotics) can synergistically enhance GLP-1 RA efficacy or mitigate weight regain after drug discontinuation.

Only after successful validation across these sequential stages could microbiome-guided precision endocrinology become a realistic and actionable clinical objective.

## Conclusion

Research over the last decade has advanced understanding of the potential interplay between GLP-1 RAs and the gut microbiota. Beyond established endocrine actions, GLP-1 RAs have frequently been associated with remodeling of the intestinal ecosystem toward configurations linked to metabolic improvement. Emerging data suggest that baseline microbiota features may be associated with variability in therapeutic response; however, current human evidence remains limited, heterogeneous, and largely associative. Robust longitudinal and interventional studies will be required before microbiome-informed therapeutic strategies can be implemented in clinical practice. A precision-oriented perspective integrating hormonal pharmacotherapy with microbiota modulation may represent a potential route toward more durable metabolic benefits and individualized obesity care, but will require standardized analytics and causality-focused trials before clinical translation. If validated, integration of microbial profiling with incretin-based therapy may contribute to more individualized approaches for obesity and T2D management.
